# Centers and peripheries: Spatial analysis of social inequalities in health in Antioquia, 2017–2023

**DOI:** 10.1371/journal.pgph.0006857

**Published:** 2026-08-28

**Authors:** Gino Montenegro-Martínez

**Affiliations:** Dentistry Faculty, University of Antioquia, Medellín, Colombia; National Center for Chronic and Noncommunicable Disease Control and Prevention, Chinese Center for Disease Control and Prevention, CHINA

## Abstract

This study aims to analyze the performance and geographical distribution of health indicators in relation to social indicators in the municipalities of Antioquia. An ecological cross-sectional study was conducted using data on health outcomes, social indicators, and institutional capacity of the municipalities of the department for the period 2017–2023. The analysis was performed using global and local Moran’s I statistics in order to identify the spatial correlation between health and social indicators**.** The global analysis reported significant associations only for the infant mortality rate, which showed positive correlations with the Multidimensional Poverty Index (MPI), Unmet Basic Needs (UBN), and Armed Conflict Index (ACI), and a negative correlation with the Municipal Performance Index (MPIn). The local analysis revealed correlations between poorer health and social indicators concentrated mainly in municipalities in the northern of the department, including the subregions of Bajo Cauca, Urabá, and Norte, while better conditions were found in municipalities of the Valle de Aburrá and parts of eastern Antioquia. Significant health inequalities persist in Antioquia, closely linked to poverty, armed conflict, and institutional limitations. The identified spatial patterns confirm a center–periphery dynamic, highlighting the need for planning approaches that are more sensitive to territorial diversity and local trajectories.

## Introduction

Health is a complex phenomenon determined by the interaction of biological and genetic factors, as well as the social, economic, political, cultural, and environmental conditions inherent to the contexts in which individuals live, act, and interact [1]. In this sense, there is a close relationship between levels of development, poverty, employment, and other factors, and health outcomes [2]. These factors not only help explain the inequalities observed between countries but also demonstrate how they are systematically reproduced within them [3,4].

The study of the relationship between social inequalities and health outcomes is a core area of public health, aimed to analyze the health status of populations and provide evidence to inform decision-making. Various approaches have been developed for measuring and analyzing it. One approach has focused on selecting stratifiers, such as education, income, and location, to represent the socioeconomic position of the groups analyzed. This facilitates the assessment of whether health outcomes are distributed unequally based on differential susceptibility and vulnerabilities [3,5]. Findings from this approach highlight the existence of social gradients as an explanatory element of social inequalities in health [6].

Another perspective understands social inequalities in health as structural phenomena sustained by political-institutional and symbolic arrangements that regulate the distribution of power and resources in society [7]. From this standpoint, inequalities are not accidents or technical failures; they are constitutive consequences of a societal model. This approach enables the analysis of how the political organization of territory contributes to the creation of spaces that accumulate favorable conditions, while others remain subordinated and excluded [7,8].

Previous research in Antioquia has shown that mortality from external causes and cervical cancer is higher in the poorest municipalities with lower social and economic development, concentrated in the northern and western areas of the department [9,10]. These findings challenge the idea that national and departmental policies operate with homogeneous effects across the territory. On the contrary, they coexist and interact with other forms of power—economic, ideological, and military [11]—that shape relations of conflict, alliances, support, or opposition [12], upon which their effectiveness depends.

This perspective supports the argument that health inequalities are not explained solely by individual factors, group characteristics, or their socioeconomic positions (e.g., income, educational level), but also by the local configuration of political power and state action. Such an approach is particularly relevant in Colombia, a country marked by profound territorial heterogeneity rooted in historical processes of spatial appropriation and resource control dating back to the colonial period [13].

These trajectories have led to persistent inequalities between regions and municipalities, as evidenced by their differential capacity to articulate economic, social, and political projects [14]. As a result, a dynamic of centers and peripheries emerges, structuring power relations across the territory [15,16]. In the centers, higher levels of institutionalization and provision of public goods are consolidated, while the peripheries become spaces where extractive political institutions prevail, concentrating power and limiting state capacity [15]. This scenario facilitates the reproduction of poverty and social exclusion. In addition, it combines with the dynamics of violence associated with the presence of illegal armed groups, creating a context in which social inequalities in health not only persist but are systematically deepened and territorialized [12].

At the global level, calls have been made to generate evidence that can contribute to addressing social health inequalities [17]. Furthermore, the importance of incorporating the geographic dimension into the analysis of health inequalities has been recognized [18]. However, we did not identify studies specifically conducted in Antioquia that aimed to analyze the performance of social and health indicators from a spatial perspective, focusing on social health inequalities. Therefore, the purpose of this study is to analyze the performance and geographic distribution of health indicators in relation to social indicators across the municipalities of Antioquia.

## Methods and materials

**Fig 1:**
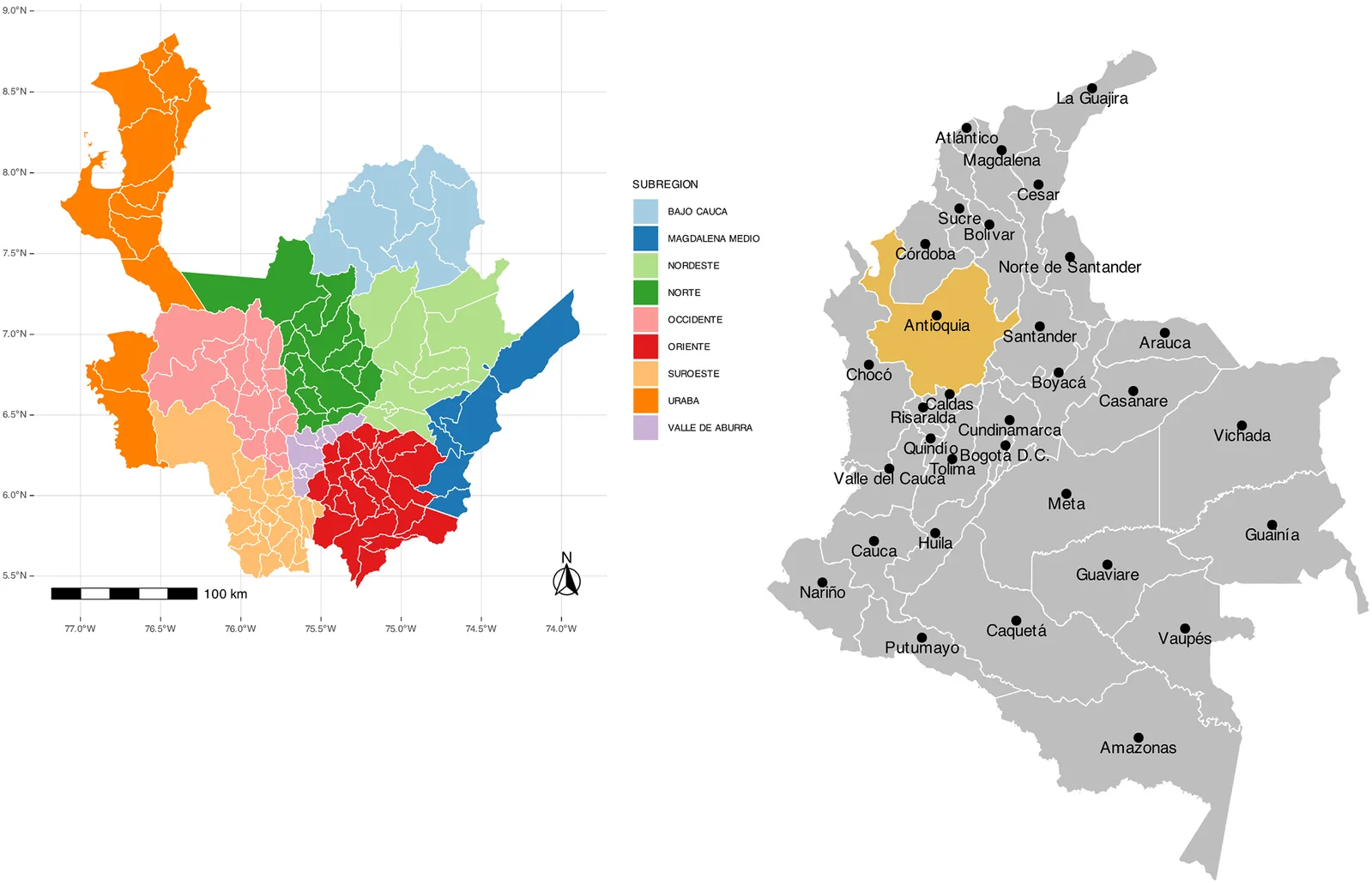
Colombia by departments and Antioquia by subregions. Source: Municipal boundary shapefile obtained from Datos Abiertos Antioquia, Government of Antioquia, Colombia [21]. Thematic map created by the author in **R.**

Antioquia is one of Colombia’s 32 departments, comprising 125 municipalities organized into nine subregions (Fig 1). It borders the department of Córdoba to the north, Chocó to the west, Risaralda and Caldas to the south, and the Magdalena River Valley to the east, where the departments of Boyacá, Santander, and Bolívar are located [19]. The projected population for 2025 is 6,928,372, of whom 78.6% reside in urban areas. Medellín is the capital city, concentrating 36.4% of the department’s population [20].

This study employed a cross-sectional ecological design, examining health outcomes, social indicators, and measures of institutional capacity across the 125 municipalities of Antioquia between 2017 and 2023. Health indicators were obtained from the Virtual Health Information Platform for Research, provided by the Departmental Secretariat of Health and Social Protection of Antioquia [22]. The following indicators were constructed: (a) infant mortality rate per 1,000 live births; (b) perinatal mortality rate per 1,000 live births; (c) under-5 mortality rate per 1,000 live births; and (d) maternal mortality ratio per 100,000 live births.

The social indicators included are the Multidimensional Poverty Index (MPI) at the municipal level, as reported in the 2018 Census, available from the National Administrative Department of Statistics [23]. A second measure was the Armed Conflict Intensity Index (ACI) at the municipal level for the 2017–2023 period, obtained from the Subdirectorate for Human Rights and Peace of the National Planning Department. This index synthesizes armed actions, homicides, kidnappings, landmine victims, forced displacement, and coca cultivation, and additionally incorporates the homicide of social leaders and ex-combatants [24]. The third indicator was the Municipal Performance Index (MPIn) for the period 2017–2022, as reported by the Directorate of Territorial Planning of the National Planning Department. This indicator assesses local government management and the achievement of development outcomes (i.e., population quality of life) [25]. Finally, the Unmet Basic Needs (UBN) indicator was retrieved from the 2018 Census, also provided by the National Administrative Department of Statistics [26].

The analysis was conducted at the municipal level using the Global and Local Bivariate Moran’s I (LISA), incorporating the selected health and social variables. The Global Moran’s I measures the degree of spatial correlation between the values of two variables across the study territory, thus identifying whether there is a general trend of geographic units with similar values (high or low) clustering spatially. This index ranges from -1–1, positive values indicate a positive correlation and spatial clustering of the two variables, negative values indicate a negative correlation, and values close to 0 indicate absence of correlation [27].

In turn, LISA decomposes the Global Moran’s I to identify the precise location of spatial patterns. Each municipality is classified according to its relationship with neighbors, distinguishing between high-high clusters (municipalities with high values in a health indicator surrounded by high values in the social indicator), low-low clusters (low values surrounded by low values), as well as spatial dissonance patterns (high-low and low-high).

To calculate each index the spatial dependence was modeled using the queen contiguity criterion, constructed from the polygon geometry of territorial units. A row-standardized spatial weights matrix was generated, allowing for units without neighbors, ensuring each row summed to 1 and facilitating the interpretation of spatially lagged means.

For the Global Bivariate Moran’s I, the observed statistic was compared against a distribution of random permutations to determine the statistical significance of the spatial pattern. Inference was based on a two-tailed p-value, calculated as the proportion of simulations in which the absolute index was equal to or greater than the empirically estimated value. Results are summarized in a table reporting, for each health indicator, the Moran statistic identifying the direction and strength of the spatial correlation between variables and its significance with p-value.

For the LISA, local clusters were classified according to the standardized value of the health variable (zₓ) and the spatial lag (weighted average of neighbors) of the social variable (lag zᵧ), distinguishing areas of positive spatial association (high-high, low-low) from those of negative association (high-low, low-high). The classification criteria were as follows:

- High–High: zₓ > 0 and lag zᵧ > 0 with p < 0.05 = municipality with high health values surrounded by municipalities with high social values.
- Low–Low: zₓ < 0 and lag zᵧ < 0 with p < 0.05 = municipality with low health values surrounded by municipalities with low social values.
- High–Low: zₓ > 0 and lag zᵧ < 0 with p < 0.05 = municipality with high health values surrounded by neighbors with low social values (outlier).
- Low–High: zₓ < 0 and lag zᵧ > 0 with p < 0.05 = municipality with low health values surrounded by neighbors with high social values (outlier).
- Not significant: p ≥ 0.05.

Bivariate clusters were represented in maps using a discrete color palette for the five categories, along with a table including the municipality, cluster category, original values of both variables, the local statistic (Ib), and the p-value (S1 Data). The Shapefile used to elaborate maps was downloaded from Datos Abiertos Antioquia [21]. All analyses and maps were performed in RStudio version 2025.05.1 + 513 (open-source license), using, among others, the following packages: sf for handling geometries, spdep/sfdep for spatial inference (neighbors, weights, and bivariate Moran’s I), ggplot2 for visualization, and writexl for exporting tables.

## Results

The analysis of the global bivariate Moran’s I revealed statistically significant spatial correlations only for the infant mortality rate in relation to the selected social variables. A positive spatial correlation was identified with the MPI (I = 0.316; p < 0.001), the ACI (I = 0.266; p < 0.001), and UBN (I = 0.396; p < 0.001). In contrast, a negative spatial correlation was observed with the MPIn (I = –0.258; p < 0.001), suggesting that municipalities with low institutional performance tend to be located near municipalities with high infant mortality, and vice versa. No statistically significant global spatial associations were found for the other health indicators with the social variables considered (Table 1).

**Table 1:** Global bivariate Moran’s I between health and social indicators in the municipalities of Antioquia.

Health indicator	Social indicator	Global Moran’s I	*p*
Infant mortality rate per 1,000 live births	MPI	0,316	*0,00*
	MPIn	-0,258	*0,00*
	CAI	0,266	*0,00*
	UBN	0,396	*0,00*
Under-5 mortality rate per 1,000 live births	MPI	-0,026	*0,52*
	MPIn	-0,055	*0,21*
	CAI	-0,051	*0,10*
	UBN	-0,033	*0,33*
Maternal mortality ratio per 100,000 live births	MPI	0,001	*0,97*
	MPIn	-0,058	*0,20*
	CAI	-0,018	*0,59*
	UBN	0,001	*0,98*
Perinatal mortality rate per 1,000 live births	MPI	-0,043	*0,35*
	MPIn	-0,050	*0,28*
	CAI	-0,050	*0,14*
	UBN	-0,060	*0,13*

Source: Author elaboration.

**Fig 2:**
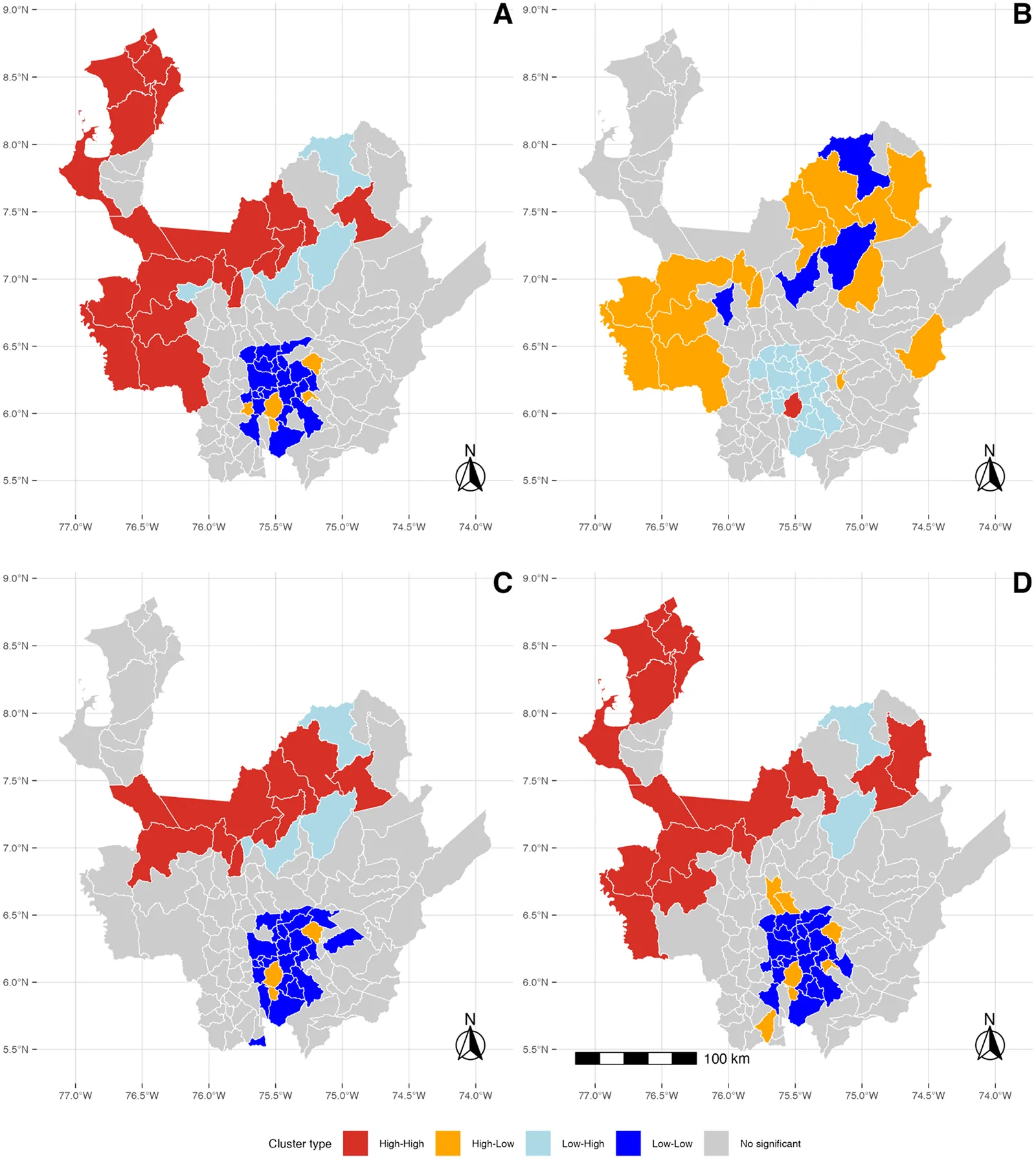
Spatial correlation between the infant mortality rate and selected social indicators. **(A)** Infant mortality rate and Multidimensional Poverty Index; **(B).** Infant mortality rate and Municipal Performance Index; **(C).** Infant mortality rate and Armed Conflict Intensity Index; **(D)** Infant mortality rate and Unmet Basic Needs. Source: Municipal boundary shapefile obtained from Datos Abiertos Antioquia, Government of Antioquia, Colombia [21]. Thematic map created by the author in **R.**

The LISA analysis identified consistent geographic patterns between the infant mortality rate and the selected social variables. In relation to MPI, UBN, and ACI, high–high clusters were concentrated mainly in municipalities located in the northern part of the department, including those grouped in the Urabá, Norte, and Occidente regions. Conversely, low–low clusters were in the Valle de Aburrá and parts of the eastern subregion. High–low and low–high clusters were less frequent and more dispersed. Regarding the MPIn, the spatial correlation was negative. High–low clusters were primarily located in Bajo Cauca and Urabá, while low–high clusters were concentrated in the Valle de Aburrá and the eastern subregion (Fig 2).

**Fig 3:**
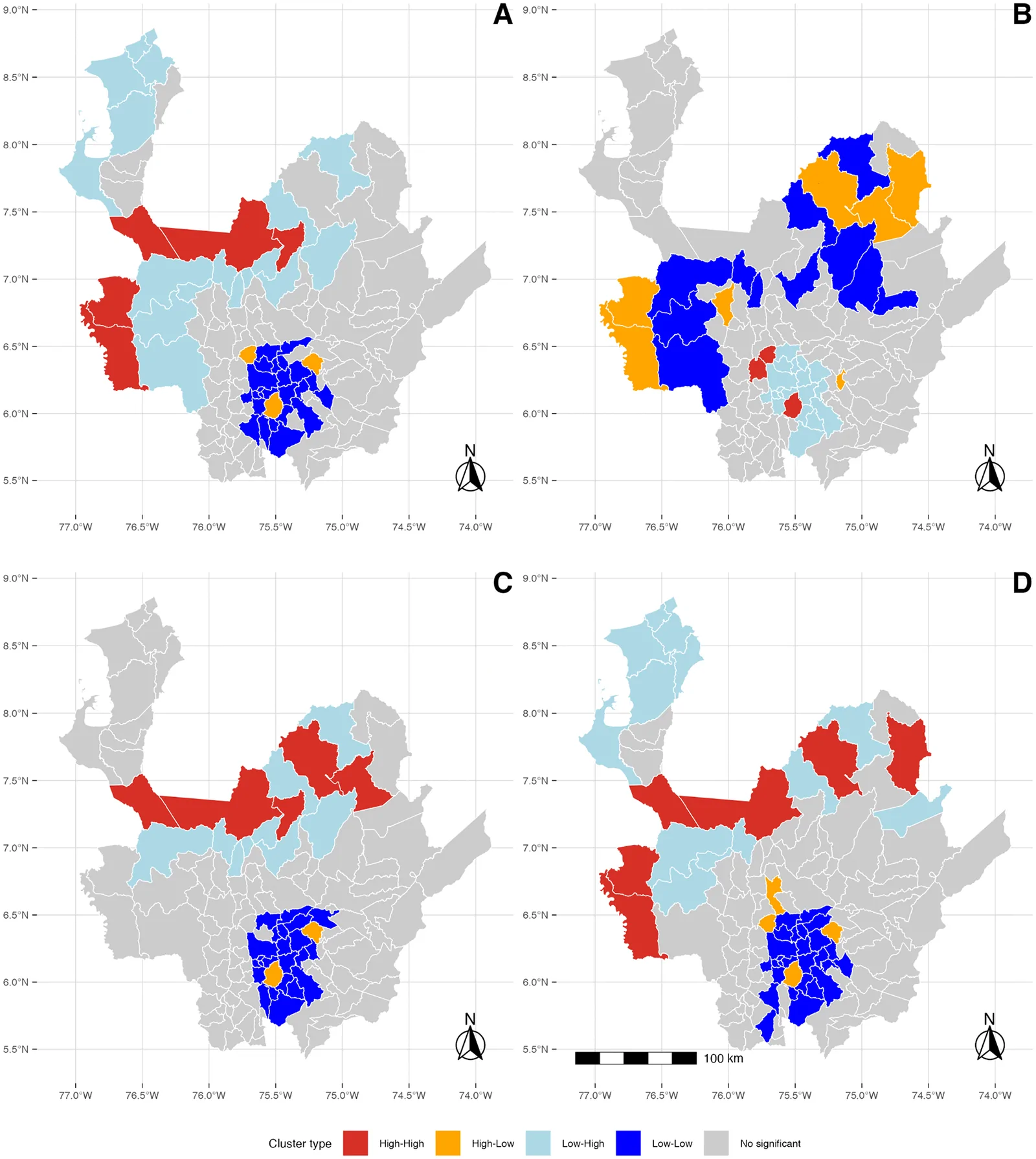
Spatial correlation between the under-5 mortality rate and selected social indicators in the municipalities of Antioquia. **(A)** Under-5 mortality rate and Multidimensional Poverty Index; **(B)** Under-5 mortality rate and Municipal Performance Index; **(C)** Under-5 mortality rate and Armed Conflict Intensity Index; **(D)** Under-5 mortality rate and Unmet Basic Needs. Source: Municipal boundary shapefile obtained from Datos Abiertos Antioquia, Government of Antioquia, Colombia [21]. Thematic map created by the author in R.

For the under-5 mortality rate, the comparisons with MPI, UBN, and ACI showed a pattern that partially differed from that observed for infant mortality. Although high–high clusters were more dispersed, a concentration of worse health outcomes along with neighboring municipalities with worse social indicators was evident in the northern part of the department, particularly in Bajo Cauca, Urabá, and some areas of Norte. Low–low clusters, similar to those in infant mortality, were primarily located in the Valle de Aburrá and the eastern subregion. High–low and low–high clusters appeared more heterogeneously (Fig 3).

**Fig 4:**
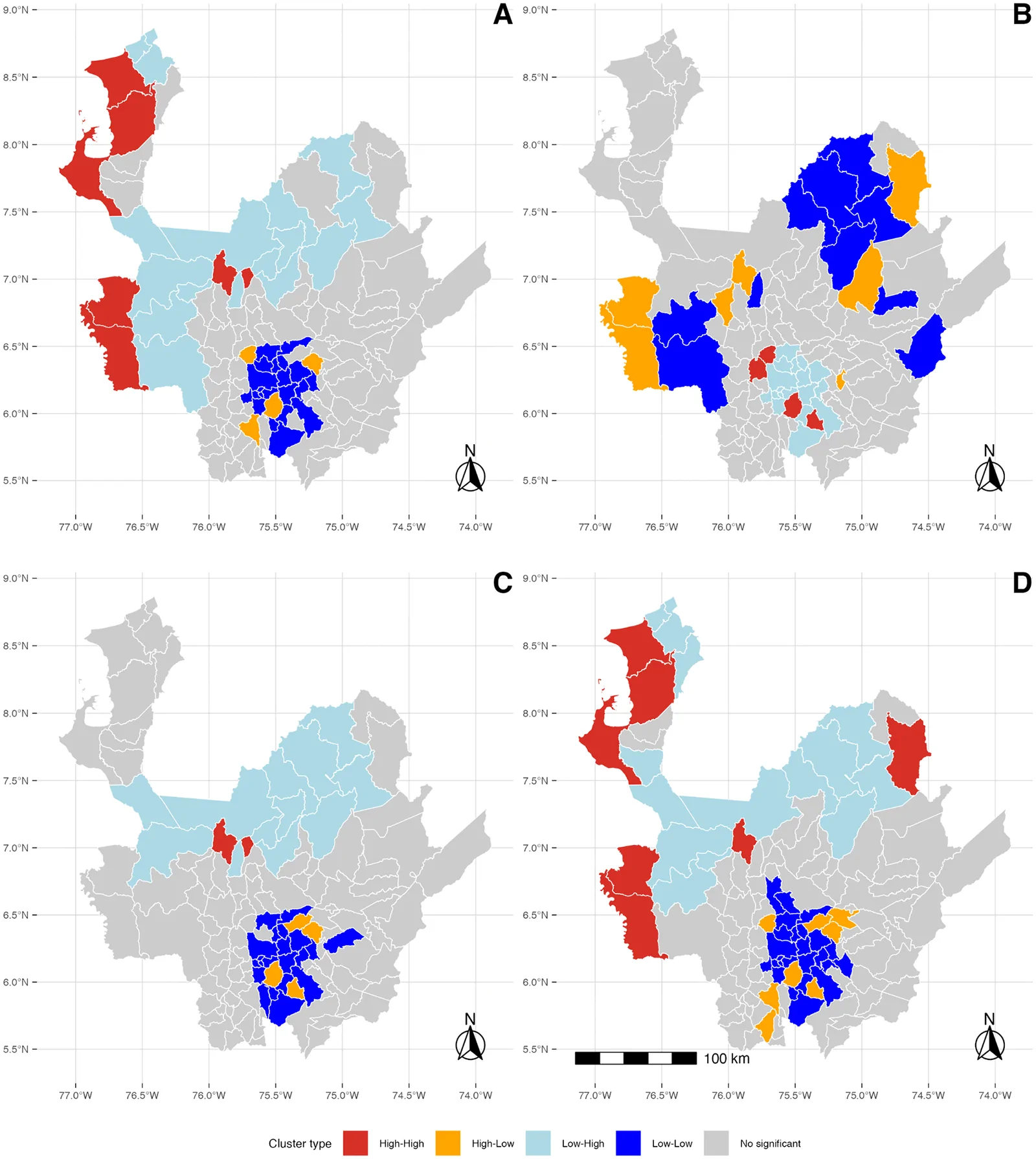
Spatial correlation between the maternal mortality ratio and selected social indicators in the municipalities of Antioquia. **(A)** Maternal mortality ratio and Multidimensional Poverty Index; **(B)** Maternal mortality ratio and Municipal Performance Index; **(C)** Maternal mortality ratio and Armed Conflict Intensity Index; **(D)** Maternal mortality ratio and Unmet Basic Needs. Source: Municipal boundary shapefile obtained from Datos Abiertos Antioquia, Government of Antioquia, Colombia [21]. Thematic map created by the author in **R.**

For the maternal mortality ratio, spatial patterns were less consistent than those observed for infant and under-5 mortality. However, they shared a concentration of worse health outcomes with neighboring municipalities exhibiting poorer social indicators in Urabá and Bajo Cauca. Low–low clusters were mainly located in the Valle de Aburrá and some municipalities in the eastern subregion. Regarding the MPIn, high–low clusters were observed in municipalities in Urabá and Nordeste, while low–high clusters were in the Valle de Aburrá. High–low and low–high clusters were generally less frequent and more dispersed (Fig 4).

**Fig 5:**
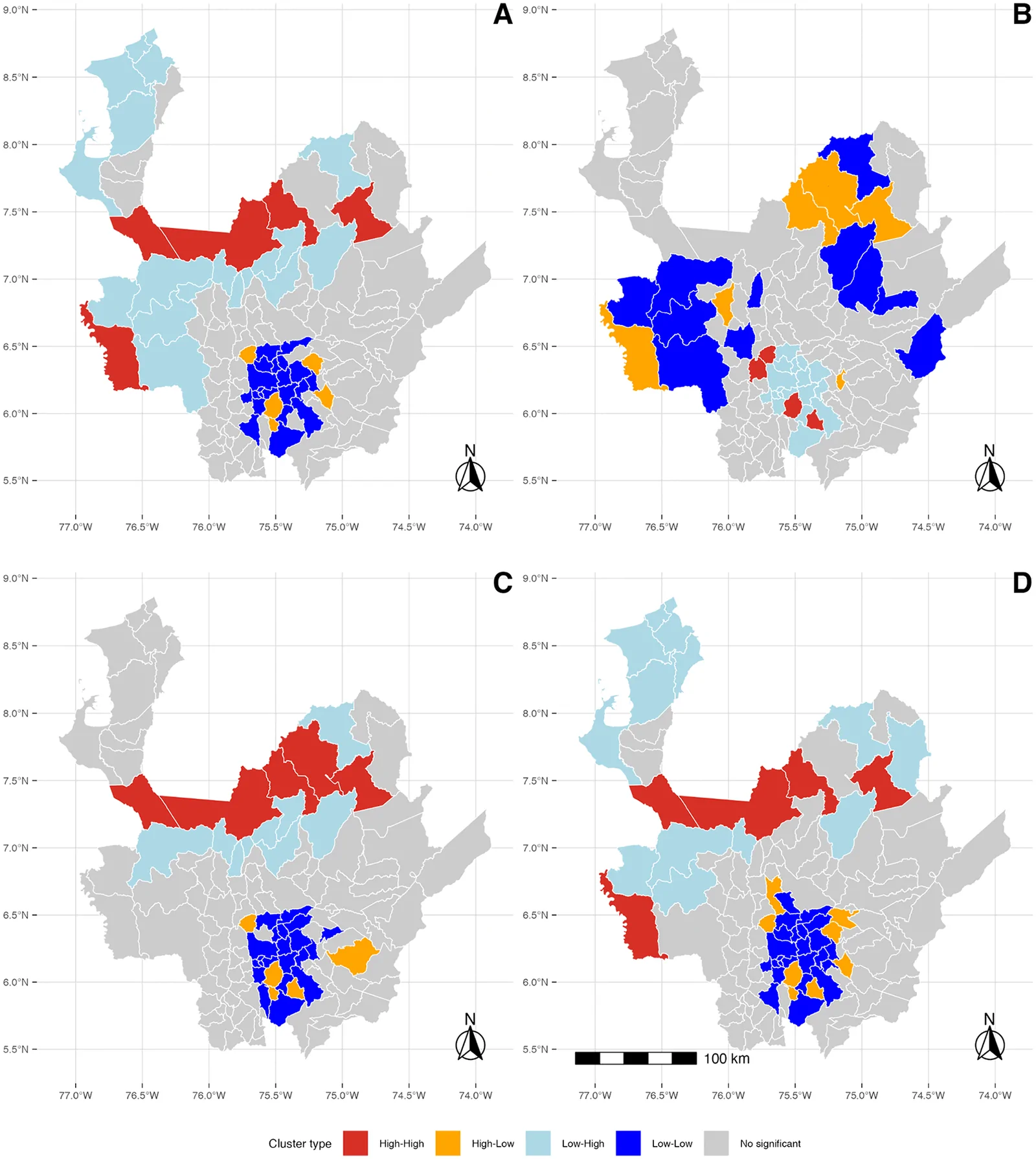
Spatial correlation between the perinatal mortality rate and selected social indicators in the municipalities of Antioquia. **(A)** Perinatal mortality rate and Multidimensional Poverty Index; **(B)** Perinatal mortality rate and Municipal Performance Index; (C) Perinatal mortality rate and Armed Conflict Intensity Index; (D) Perinatal mortality rate and Unmet Basic Needs. Source: Municipal boundary shapefile obtained from Datos Abiertos Antioquia, Government of Antioquia, Colombia [21]. Thematic map created by the author in **R.**

For the perinatal mortality rate, isolated high–high clusters were identified in municipalities of Bajo Cauca, Urabá, and Norte, where high perinatal mortality coincided with neighbors showing high levels of poverty (UBN, MPI) or greater conflict intensity (ACI). As in the previous results, low–low clusters were mainly located in the Valle de Aburrá and some municipalities in the eastern subregion. In relation to the MPIn, some high–low clusters were observed in Bajo Cauca and Urabá, while low–high clusters were observed in areas of the Valle de Aburrá (Fig 5).

## Discussion

This study aimed to analyze the performance and geographic distribution of health indicators in relation to social indicators across the municipalities of Antioquia. Two main findings emerged. First, the global analysis reported significant associations only for the infant mortality rate, which showed positive correlations with the MPI, UBN, and ACI, and a negative correlation with the MPIn. Second, the local analysis revealed correlations between poorer health and social indicators concentrated mainly in municipalities in the northern of the department, including the subregions of Bajo Cauca, Urabá, and Norte, while better conditions were found in municipalities of the Valle de Aburrá and parts of eastern Antioquia.

Colombia has historically been marked by profound inequalities, a condition that constitutes both the starting point and the primary motivation for this study. Recent analyses of these inequalities have sought a more dynamic understanding by recognizing the complexity of historical, social, racial, economic, and environmental processes that have shaped current territorial asymmetries [7]. Within this framework, the forms of spatial production and appropriation, disputes over power at the local, regional, and national levels, and the armed conflict are part of the explanatory factors. Thus, health disparities not only reflect social and economic differences but also reveal the persistence of a political order that reinforces poverty, exclusion, and weak governance in peripheral areas, thereby deepening the reproduction of social inequalities in health [7,28]. This perspective guided the discussion of our results.

Municipalities with higher infant mortality rates are near to municipalities with higher ACI values, suggesting that the persistence of armed conflict may shape conditions that adversely affect child survival. In Colombia, armed conflict has been a longstanding feature of the country’s history and, since the 1960s, has involved multiple actors and dynamics that have generated profound social and humanitarian consequences, including displacement, confinement, violence, land dispossession, and the deterioration of living conditions [29].

In this context, children represent one of the populations most vulnerable to both the direct and indirect effects of conflict [30]. Armed conflict can disrupt public health programs, limit institutional presence, and hinder access to essential services such as healthcare, potable water, sanitation, and adequate nutrition [31]. Consequently, child mortality in conflict-affected territories may also reflect broader structural conditions frequently associated with these settings, including weak state infrastructure, food insecurity, forced displacement, and restricted access to quality health services [30]. Previous studies conducted in Colombia have documented that armed conflict was associated with substantial increases in child mortality [30], while similar patterns have also been reported in African countries, reinforcing the plausibility of this relationship [32].

Municipalities with higher infant mortality rates also tended to present lower municipal institutional capacity, suggesting that local governance conditions may influence the ability of health systems to respond effectively to population needs. Decentralisation reforms were originally promoted under the assumption that transferring responsibilities and fiscal autonomy to local governments would improve access to health services and strengthen health outcomes by enabling authorities to allocate resources according to local priorities and contextual needs. In theory, local governments are better positioned to identify vulnerable populations and implement interventions adapted to territorial realities [33]. However, the effectiveness of decentralisation depends largely on the institutional capacities available at the municipal level, including administrative, technical, organizational, financial, and human resources [33,34]. Previous study in Colombia shown fiscal decentralisation is related with a decreased of infant mortality; this reduction was higher in non-poor municipalities than in poor municipalities. In this context, limited institutional capacity may reduce the ability of municipalities to sustain preventive and healthcare services essential for maternal and child health. Conversely, greater fiscal decentralisation and accumulated local experience in managing health resources are associated with reductions in infant mortality, particularly in municipalities with stronger institutional and economic conditions, supporting the idea that effective local governance can contribute to better health outcomes [34].

Municipalities with higher levels of poverty, measured through indicators such as the Multidimensional Poverty Index (MPI) and unmet basic needs (UBN), also exhibited higher infant mortality rates, suggesting that socioeconomic deprivation continues to shape unequal opportunities for child survival in Colombia. Poverty, unmet basic needs, female illiteracy, and rurality are associated with limited access to essential services and resources, contributing to poorer health outcomes and reinforcing social inequalities. These interconnected socioeconomic and demographic determinants create conditions that increase the risk of avoidable childhood mortality, particularly in territories where populations face barriers to adequate nutrition, education, housing, sanitation, and timely healthcare services [35,36].

Although Colombia experienced a substantial decline in avoidable under-five mortality during the first decades of the 21st century, this reduction was uneven across territories, revealing persistent inequities linked to structural determinants such as poverty, maternal education, and unequal access to health services. [36]. In this context, territory itself operates as a social determinant of health, as spatial and socioeconomic inequalities influence the distribution of resources and the real possibilities for populations to achieve well-being. Moreover, territorial disparities in institutional development, fiscal capacity, and the effects of armed conflict may further limit local governments’ ability to address social needs effectively [30,31,34–36]. Previous studies have also documented significant inequalities in infant mortality according to maternal educational level, health insurance enrolment, and area of residence, reinforcing the idea that child mortality is strongly conditioned by broader social and territorial inequalities [35,36].

The remaining health indicators analyzed: under-five mortality, maternal mortality, and perinatal mortality, did not show significant global spatial associations with the social indicators included in the study. One possible explanation is that these outcomes may be influenced by a more complex combination of factors that are not fully captured by broad structural indicators such as poverty, unmet basic needs, or armed conflict intensity. Previous studies have shown that maternal and child mortality are shaped not only by poverty, but also by factors such as access to healthcare, geographical barriers, maternal education, sanitation, nutrition, and the quality of health systems [37,38]. In contrast, infant mortality tends to reflect cumulative social and environmental exposures operating throughout early life, making it more sensitive to structural inequalities related to poverty, institutional capacity, and territorial exclusion. Furthermore, the absence of significant global associations does not necessarily imply that social inequalities are irrelevant for these outcomes, but rather that their spatial distribution may be more heterogeneous across municipalities, without exhibiting a clear and consistent territorial clustering pattern at the departmental level.

In the Colombian case, and particularly in Antioquia, this implies that health and social outcomes cannot be reduced merely to technical failures, infrastructural deficiencies, or lack of political will. Instead, as discussed, they reflect the concrete conditions of a territory configured as a contested space of power among political parties, armed groups, and other local forces [12]. This situation highlights, as Mann [11] argued, that not all states have successfully monopolized legitimate coercion or deployed their authority uniformly. In many cases, such monopolies have been only partial and highly differentiated depending on geographic space and local power relations—an essential consideration when analyzing health outcomes.

Spatial clusters characterized by the coexistence of poorer health and social outcomes were concentrated mainly in the northern and northeastern municipalities of Antioquia, whereas clusters with more favorable indicators were predominantly located in the Valle de Aburrá and parts of the eastern subregion. This spatial pattern, consistently observed across the maps presented in this study, suggests a territorial organization of inequalities in which adverse social and health conditions tend to cluster geographically in peripheral areas of the department.

This finding can be explained as the result of a departmental center–periphery dynamic. Such dynamics are not only spatial arrangements but also relate to imaginaries that assign symbolic and functional hierarchies to particular territories, shaping political geographies. In this context, peripheries are conceived as subordinate spaces or as inhabited by people with fewer capabilities, whose development must align with models imposed from the center [13]. This scheme reinforces territorial subordination by imposing logics that disregard local trajectories, thereby deepening center–periphery inequalities and consolidating marginalization processes with historical roots [39].

The center–periphery relationship also reflects the presence of extractive political institutions, which are more prevalent in peripheral areas [15]. These institutions promote power concentration and limited state provision, resulting in the unequal distribution of political and economic institutions, the persistence of poverty, and lower levels of development [15,16]. Manifestations of this include uncompetitive and clientelist elections. In 2022, municipalities in Bajo Cauca, Nordeste, Norte, and Urabá reported threats and vulnerabilities indicative of electoral fraud and violence, coinciding with worse social and health outcomes [40].

The configuration of centers and peripheries is also linked to violence [39]. Antioquia has been historically affected by violence, though this phenomenon is more frequent in specific municipalities. For instance, Urabá, Bajo Cauca, Norte, and Oriente have recorded the highest number of displaced communities. At the same time, municipalities in Bajo Cauca, such as El Bagre, Nechí, Caucasia, Cáceres, and Tarazá, report the highest numbers of land dispossessions [41]. Likewise, 50% of applications to the Registry of Forcibly Dispossessed and Abandoned Lands in Antioquia are concentrated in Urabá (29.1%), Bajo Cauca (15.7%), and Nordeste (5.5%) [42].

Regarding armed group presence, between 2020 and 2021, paramilitary incursions were recorded in Bajo Cauca, Oriente, Nordeste, and Suroeste, in addition to a massacre in Caucasia (Bajo Cauca) in 2021 [43]. Currently, Bajo Cauca, Norte, and Nordeste are disputed by the Clan del Golfo and factions of the Estado Mayor Central, in alliance with the Ejército de Liberación Nacional (ELN). In parallel, the Clan del Golfo dominates Urabá and parts of Bajo Cauca [44]. Following the Peace Agreement with the Fuerzas Armadas Revolucionarias de Colombia- Ejército del Pueblo (FARC-EP), new armed actors have expanded and consolidated, affecting regions such as Bajo Cauca with increased homicides and kidnappings [45].

From an international perspective, our findings align with Centeno [46], who argued that Latin American states historically developed as weak, fragmented, and limited in their capacity to guarantee rights. The inequalities observed in Antioquia are therefore not an isolated phenomenon but part of a broader regional trajectory. Similar patterns have been identified in Brazil [47], Ecuador [48], and Mexico [49], illustrating geographic patterns linked to social inequalities in health. This study thus provides evidence from a subnational case that connects with global debates on the state in society [12] and contributes to understanding the persistence of health inequalities from a geographic perspective [18]. Also contributes to the knowledge where armed conflict is related with poorer health outcomes [50].

The findings of this study help elucidate the geographic distribution of health inequalities in Antioquia and their relationship with social and institutional conditions. Identifying local clusters within a center–periphery pattern provides valuable input for guiding interventions that prioritize areas where poorer health outcomes coexist with greater social deprivation. Furthermore, the finding that observed patterns do not align with the current subregional division highlights the need to revise territorial planning criteria, incorporating the spatial dimension of inequalities as a strategic input for public policy design and intersectoral coordination in health.

Additionally, this analysis is particularly useful for understanding why certain municipalities in Antioquia consistently present poorer health, social, and institutional outcomes. These are not simply geographically remote territories but peripheries that, according to this study, remain excluded from effective rights guarantees.

This study has the limitations inherent to ecological designs, where observed associations apply at the aggregate level and cannot be directly extrapolated to individuals (ecological fallacy). Moreover, secondary data sources were used, which, although official, may contain underreporting or variations in data quality by municipality and indicator. The cross-sectional nature of the analysis precludes causal inference; thus, the identified spatial patterns should be interpreted as territorial associations valid for the study period. Finally, the selection of social and health indicators was constrained by the availability and comparability of municipal-level data, which excluded other potentially relevant factors that could explain the observed inequalities.

## Conclusions

Among the indicators analyzed, infant mortality showed the strongest association with structural social conditions, exhibiting positive spatial correlations with the MPI, UBN, and the ACI, as well as a negative correlation with MPIn. Poorer social and health indicators were concentrated mainly in the northern subregions, particularly Bajo Cauca and Norte, while more favorable conditions were observed in the Valle de Aburrá and some municipalities of eastern Antioquia, reflecting a territorial dynamic structured around relations of center-periphery.

These findings suggest that infant mortality cannot be understood solely as a health outcome, but rather as the expression of historically configured socioeconomic, political, and institutional inequalities that shape unequal opportunities for survival and well-being across territories. The concentration of poorer health and social indicators in the peripheral municipalities of northern Antioquia, contrasted with the more favorable conditions observed in the departmental center, reflects a center–periphery dynamic in which poverty, armed conflict, institutional fragility, and uneven state presence converge to reproduce territorial inequalities in health.

## Supporting information

S1 Data Excel file containing the municipal-level data and spatial analysis outputs used in this study. The file includes different worksheets (WS) describing local spatial autocorrelation analyses conducted between health indicators and social indicators across municipalities in Antioquia, Colombia. **WS1_Dataset.** Database used for the global and local spatial analysis of health and social indicators across municipalities in Antioquia, Colombia. The dataset includes municipal-level health and social indicators. **WS2_im_mpi.** Local spatial autocorrelation analysis between infant mortality rate and the Multidimensional Poverty Index. **WS3_im_mpin.** Local spatial autocorrelation analysis between infant mortality rate and municipal institutional capacity. **WS4_im_cai.** Local spatial autocorrelation analysis between infant mortality rate and the Armed Conflict Index. **WS5_im_ubn.** Local spatial autocorrelation analysis between infant mortality rate and unmet basic needs. **WS6_u5_mpi.** Local spatial autocorrelation analysis between under-five mortality rate and the Multidimensional Poverty Index. **WS7_u5_mpin.** Local spatial autocorrelation analysis between under-five mortality rate and municipal institutional capacity. **WS7_u5_cai.** Local spatial autocorrelation analysis between under-five mortality rate and the Armed Conflict Index**. WS8_u5_ubn.** Local spatial autocorrelation analysis between under-five mortality rate and unmet basic needs. **WS9_mm_mpi.** Local spatial autocorrelation analysis between maternal mortality ratio and the Multidimensional Poverty Index. **WS10_mm_mpin.** Global and local spatial autocorrelation analysis between maternal mortality ratio and municipal institutional capacity. **WS11_mm_cai.** Local spatial autocorrelation analysis between maternal mortality ratio and the Armed Conflict Index. **WS12_mm_ubn.** Local spatial autocorrelation analysis between maternal mortality ratio and unmet basic needs. **WS13_mm_mpi.** Local spatial autocorrelation analysis between perinatal mortality rate and the Multidimensional Poverty Index. **WS14_pr_mpin.** Local spatial autocorrelation analysis between perinatal mortality rate and municipal institutional capacity. **WS15_pr_cai.** Local spatial autocorrelation analysis between perinatal mortality rate and the Armed Conflict Index. **WS16_pr_unb.** Local spatial autocorrelation analysis between perinatal mortality rate and unmet basic needs.
(XLSX)
